# Spatiotemporal epidemiology and associated risk factors of tuberculosis incidence and mortality in Indonesia 2017–2022: a nationwide space-time hierarchical analysis

**DOI:** 10.1186/s12963-026-00458-5

**Published:** 2026-01-31

**Authors:** Abdillah Farkhan, Tiffany Tiara Pakasi, Sulistyo Sulistyo, Alya Salsabila, Richard James Maude, Chawarat Rotejanaprasert

**Affiliations:** 1 Office of the Health Quarantine I Tarakan, Directorate General of Disease Control, Ministry of Health, Tarakan, Indonesia; 2 Center for the Health System and Strategy, Ministry of Health, Jakarta, Indonesia; 3 Department of Tropical Hygiene, Faculty of Tropical Medicine, Mahidol University, Bangkok, Thailand; 4 National Tuberculosis Program, Directorate General of Disease Control, Ministry of Health, Jakarta, Indonesia; 5 Mahidol-Oxford Tropical Medicine Research Unit, Faculty of Tropical Medicine, Mahidol University, Bangkok, Thailand; 6 Centre for Tropical Medicine and Global Health, Nuffield Department of Medicine, University of Oxford, Oxford, UK

**Keywords:** Spatiotemporal, Tuberculosis, Indonesia, Incidence, Mortality, Socioeconomic, Health system

## Abstract

**Background:**

Indonesia is the second-highest contributor to global tuberculosis (TB) cases, accounting for 10% of the total. While previous studies have explored TB patterns in specific regions, a comprehensive nationwide analysis at a fine spatial scale is lacking. This study investigated spatiotemporal patterns of TB incidence and mortality, identified geographical hotspots, and examined their association with risk factors to inform public health policy.

**Methods:**

This retrospective study analyzed notified TB cases and deaths during treatment from Indonesia’s National Tuberculosis Surveillance System across 514 districts between 2017 and 2022. Spatiotemporal Bayesian hierarchical modeling was employed to identify high-risk areas and assess associations with potential risk factors. The best-fitting model was determined by evaluating various spatial and temporal random effect structures and likelihood assumptions.

**Results:**

TB incidence fluctuated with a trough during the COVID-19 pandemic and an overall increase, while mortality increased over time. Incidence hotspots clustered in urbanized areas, while mortality hotspots were scattered across the country. The best-fitting model to estimate risk factors for both outcomes was Poisson likelihood. This indicated that TB incidence was spatiotemporally positively linked to better healthcare access (RR: 1.016; 95% CI: 1.007–1.025) and higher municipal human development index (MHDI, RR: 1.062; 95% CI: 1.049–1.075). Mortality was associated with low treatment coverage (RR: 0.610; 95% CI: 0.552–0.674) and success rates (RR: 0.595; 95% CI: 0.491–0.721).

**Conclusions:**

Fluctuating TB incidence, hotspots concentrated in urbanized areas with better healthcare access and higher MHDI as well as increasing mortality linked to poor treatment outcomes underscore the need for targeted public health interventions to expand access to care, improve treatment adherence, and address the socioeconomic disparities driving TB mortality.

**Supplementary Information:**

The online version contains supplementary material available at 10.1186/s12963-026-00458-5.

## Introduction

Tuberculosis (TB), caused by *Mycobacterium tuberculosis*, remains a major global public health challenge. Transmission occurs primarily through airborne spread when individuals with active TB cough, releasing infectious particles. It is estimated that nearly one-quarter of the world’s population harbors latent infection [1]. In 2022, TB caused approximately 1.3 million deaths globally, nearly double that of HIV/AIDS, making it the second leading infectious cause of death after COVID-19 [2]. Thirty high-burden countries accounted for 87% of all new TB cases, with Indonesia ranking second, contributing 10% of the global TB burden [2]. Alarmingly, the recent global increase in TB cases between 2020 and 2022 was largely driven by Indonesia, Myanmar, and the Philippines, collectively adding approximately 400,000 additional cases [2].

Health system resources such as universal health coverage (UHC) and access to timely point-of-care diagnostics are critical for reducing TB-related mortality, particularly among individuals who remain undiagnosed [3]. Although Indonesia has implemented UHC, substantial barriers remain in TB care. Direct medical costs and delays due to initial visits to private providers often result in increased financial burdens and prolonged pre-diagnostic pathways [4]. Approximately half of TB-affected households in Indonesia experience catastrophic costs and income losses, highlighting significant economic barriers to accessing and completing treatment. Completing the full course of TB therapy, typically spanning 4 to 6 months of anti-TB drugs, is essential to prevent mortality, with untreated TB carrying a case fatality rate of nearly 50% [2]. Beyond healthcare access, social determinants such as poverty, inequality, and broader structural factors play a critical role in increasing TB transmission and poor health outcomes [5, 6].

Spatial epidemiology offers important insights into the geographic distribution of diseases, helping to identify high-burden areas and informing more effective, targeted, public health interventions [7]. Analytical approaches have evolved from simple mapping to more sophisticated spatial statistical methods, including cluster detection and ecological regression, which assess geographic correlations between disease outcomes and risk factors. In TB research, spatial methods have been widely utilized. For example, in Thailand, provincial-level TB data combined with Getis–Ord statistics identified high-incidence clusters along international borders [8]. In Brazil, generalized additive models (GAMs) linked TB cure rates to sociodemographic indicators [9], and in Chongqing, China, spatial scan statistics revealed significant mortality clusters [10]. These studies demonstrate the utility of spatiotemporal analyses in uncovering hidden patterns of disease burden and informing targeted interventions.

In Indonesia, however, spatiotemporal analyses of TB have remained limited in scope. Most studies have focused on local or provincial scales rather than nationwide patterns. For instance, kernel density estimation in Yogyakarta Province highlighted high-risk zones near social gathering points [11], while spatial autocorrelation methods detected TB clusters across subdistricts in Aceh Province [12]. Geographically weighted Poisson regression in Bandar Lampung City revealed spatial variability in TB risk factors [13], and other provincial studies identified associations between TB distribution and socioeconomic or environmental factors, such as poverty, housing conditions, and solar radiation [14, 15]. While valuable, these localized studies fall short of providing a comprehensive, national-level understanding necessary for guiding TB control strategies.

Hierarchical Bayesian spatial models offer a more robust framework for spatiotemporal epidemiological analysis, particularly by addressing stochastic variability and instability in areas with small populations [16]. By incorporating prior information and accounting for spatial and temporal dependencies, Bayesian methods can improve the precision of risk estimates and support stronger inference for surveillance and control planning [17]. To address these gaps, this study applies a Bayesian hierarchical spatiotemporal modeling approach to national TB surveillance data from Indonesia, focusing on both TB incidence and mortality during treatment. By identifying geographical hotspots and examining associations with treatment outcomes, health system indicators, and socioeconomic factors, this study aims to generate actionable evidence for policymakers and public health agencies. The findings are intended to support more targeted, equitable, and effective TB treatment, prevention and control strategies across Indonesia’s diverse regions.

## Methods

### Study design and data collection

We conducted a retrospective ecological analysis to investigate the spatial distribution and determinants of TB incidence and mortality across Indonesia. Secondary data were obtained from the National Tuberculosis Surveillance System (NTSS), managed by the Tuberculosis Working Group at the Indonesia Ministry of Health, covering the period from 2017 to 2022. The NTSS aggregates annual data on TB incidence and mortality at the district level, encompassing all 514 districts nationwide. In the NTSS, TB cases are diagnosed based on results from rapid molecular testing, while TB mortality is recorded as deaths occurring during the treatment completion period.

District-level potential risk factors were selected based on their relevance to TB outcomes, as identified in previous research [2, 3, 5, 6, 18, 19], and the availability of reliable nationwide data. Health system-related covariates included the number of health canters per 100,000 population (sourced from the National Statistical Agency) and the proportion of the population covered by social health insurance (obtained from the National Agency for Health Security). These variables represent aspects of Indonesia’s health system response to TB, reflecting the availability of points-of-care for TB detection and the role of universal health coverage (UHC) in supporting access to treatment.

Socioeconomic indicators, namely the municipal human development index (MHDI), poverty headcount index, and household sanitation access, were also obtained from the National Statistical Agency. These factors align with evidence from previous ecological studies, which have identified poor sanitation and socioeconomic inequalities as major determinants of TB burden in regions such as Latin America and the Caribbean [6], as well as the longstanding historical link between poverty and TB [5]. While these indicators may reflect overlapping constructs, each represents a distinct policy-relevant domain (human development, economic poverty, and environmental conditions).

For TB mortality analyses, additional covariates were included to reflect disease severity and treatment performance. District-level drug-resistant TB (DR-TB) case counts were included as a potential risk factor, based on previous studies highlighting substantial proportions of TB deaths occurring among DR-TB patients in settings such as Nigeria [18], South Africa [20], and Pakistan [21]. Moreover, indicators of first-line TB treatment performance—treatment coverage, treatment completion rate, and treatment success rate—were included, reflecting their critical role in determining TB treatment outcomes and mortality risk [2].

To assess potential multicollinearity among candidate covariates, we examined pairwise Spearman correlation coefficients across all variables (figure S1.1). Following previous literature, correlations exceeding 0.7 were considered indicative of strong collinearity warranting further consideration [22, 23]. Our data showed a relatively strong correlation between MHDI and life expectancy at birth (Spearman coefficient = 0.73). To minimize collinearity, only life expectancy was included in the mortality model as an outcome measure of population health reflecting underlying mortality patterns. This measure aligns closely with the main objectives of TB control programs, particularly the reduction of mortality, and therefore serves as an appropriate indicator for demonstrating the population impact of TB interventions. In contrast, the MHDI is retained in the incidence model because it is a broader composite indicator of socioeconomic development, which captures upstream structural conditions.

No other pairwise correlations exceeded the threshold, indicating limited risk of problematic multicollinearity. Given the hierarchical Bayesian framework, additional adjustment for residual correlation and unobserved heterogeneity was achieved through spatial, temporal, and spatiotemporal random effects. The final set of covariates included in the TB incidence and mortality models with their association measure estimates is summarized in Table 1. A comprehensive description of all candidate variables and their definitions is provided in table S1.1, along with the correlation matrix in figure S1.1 (supplementary document S1).

### Data analysis

#### Descriptive analysis and rate standardization

Temporal trends in TB incidence and mortality were visualised using line plots at both the provincial and district levels, areas with the highest averages identified and compared to the national trajectory. To account for population structure, we calculated standardized morbidity and mortality ratios ($\:{SMR}_{{c}_{it}}$ and $\:{SMR}_{{m}_{it}}$) in $\:i$ district and $\:t$ year, adjusting for differences in population sizes. We visualized yearly maps of both standardizations to observe changes in the spatial distribution. We used an indirect method of rate standardization as denoted in Eq. 1 to 4 of supplementary document 2.

$\:{SMR}_{{c}_{it}}$ was computed by dividing observed case number ($\:{O}_{{c}_{it}}$) by the offset or expected case number in district $\:i$ and year $\:t$ ($\:{E}_{{c}_{it}}$). $\:{E}_{{c}_{it}}$was calculated by multiplying the district population ($\:{P}_{it}$) by crude incidence rate $\:\frac{{\sum\:}_{t=1}^{T}{O}_{{c}_{it}}}{{\sum\:}_{t=1}^{T}{P}_{{n}_{it}}}$. A similar method was adopted to calculate $\:{SMR}_{{m}_{it}}$, where $\:{O}_{{m}_{it}}$ was the observed deaths in district $\:i$ and year $\:t$. To calculate $\:{E}_{{m}_{it}}$, the crude death rate $\:\frac{{\sum\:}_{t=1}^{T}{O}_{{m}_{it}}}{{\sum\:}_{t=1}^{T}{P}_{{c}_{it}}}$ was then calculated and multiplied by $\:{P}_{{c}_{it}}$. The populations of reference in $\:{\sum\:}_{t=1}^{T}{P}_{{n}_{it}}$ and $\:{\sum\:}_{t=1}^{T}{P}_{{c}_{it}}$ were distinguished, respectively, as national population numbers potentially exposed to the disease and all TB patients that may be at risk of death during treatment.

### Spatiotemporal hierarchical bayesian modelling and hotspot analysis of TB incidence and mortality

To identify high-risk areas and understand patterns of TB incidence and mortality over time, spatiotemporal hierarchical Bayesian modeling was adopted. This is a robust approach that incorporates smoothing techniques to address rate instability in small populations [16]. Poisson likelihood is commonly used in hierarchical modelling for count data [24–26], such as cases and deaths. Specifically, TB incidence and mortality were modelled as $\:{O}_{{c}_{it}}\:\sim\:Poisson\:\left({\mu\:}_{{c}_{it},\:}{E}_{{c}_{it}}\right)$ and $\:{O}_{{m}_{it}}\:\sim\:Poisson\:\left({\mu\:}_{{m}_{it},\:}{E}_{{m}_{it}}\right)$ where$\:\:{\mu\:}_{{c}_{it},\:}$ and $\:\:{\mu\:}_{{m}_{it}\:}$represent the mean incidence and mortality for each district and year, respectively. The mean was modelled using the natural logarithm as the canonical link, with linear predictors both $\:{\theta\:}_{{c}_{it}}$and $\:{\theta\:}_{{m}_{it}}$, incorporating an intercept $\:{\alpha\:}_{0}$, risk factors $\:\mathrm{X}\mathrm{it}\mathrm{j}$ and random effects. The supplementary document S.2 outlines the model construction, specifically in Eq. 5 to 10. While the Poisson likelihood is widely used, the Negative Binomial (NB) model was also evaluated as this accounts for overdispersion and offers more robust estimates [27]. The specifying model variants were then defined as $\:{O}_{{c}_{it}}\sim\:Negative\:Binomial\:\left({\mu\:}_{{c}_{it},\:}{E}_{{c}_{it}}\right)$ and $\:{O}_{{m}_{it}}\sim\:Negative\:Binomial\:\left({\mu\:}_{{m}_{it},\:}{E}_{{m}_{it}}\right)$.

The Besag, York, and Mollié (BYM) model is commonly used to estimate relative risks and account for spatial autocorrelation via spatial and non-spatial random effects [28, 29]. The spatially structured effect $\:{u}_{i}$ captures dependencies between neighbouring areas using a conditional autoregressive (CAR) model, while the unstructured effect $\:{v}_{i}$ accounts for other unexplained spatial variation. We also employed three different variations of temporal effects, namely random walk of order 1, or RW1 ($\:{\lambda\:}_{t}^{RW1})$, random walk of order 2, or RW2 ($\:{\lambda\:}_{t}^{RW2}$), and identically distributed time points ($\:{\lambda\:}_{t}$). The last random effect is $\:{\delta\:}_{it}$, a type I spatial-temporal interaction capturing interaction between $\:{v}_{i}$ with $\:{\lambda\:}_{t}$, with both assumed to be unstructured and uncorrelated. Precision parameters were modelled using a Log-Gamma distribution, with hyperparameters set to (1, 0.0005) for the CAR effect and (1, 0.00005) for unstructured and random walk effects. Fixed-effect coefficients followed a Gaussian distribution with mean zero and precision 0.001.

To detect areas with elevated risk, we employed exceedance probability estimation, calculating the likelihood that the relative risk $\:{\mu\:}_{it}/{E}_{it}$ in each area exceeded a predefined threshold *q*, where $\:{\mu\:}_{it}$ represents the model-predicted incidence or mortality [30]. An area was considered a hotspot if the posterior probability that $\:{\mu\:}_{it}/{E}_{it}>q\:$exceeded a specified significance threshold. Consistent with common practice, we set *q* = 1, representing the expected risk level. The exceedance probability was computed as $\:\mathrm{Pr}({\mu\:}_{it}/{E}_{it}>q)=1-\:\mathrm{Pr}\left(\frac{{\mu\:}_{it}}{{E}_{it}}\le\:q\right).\:$We set the significance level of this study at *α* = 0.05. Areas with exceedance probabilities greater than 0.95 (i.e., 1-*α*, with *α =* 0.05) were classified as statistically significant hotspots [31, 32].

### Model selection

Combinations of random effects were tested to identify the best-fitting model, including structured, unstructured, BYM and temporal effects, along with the addition of type I interaction. By incorporating various temporal components, 18 initial spatiotemporal models were developed. Through separate modelling for TB incidence and mortality under different likelihoods, a total of 72 models were constructed. Optimal models for explaining associations with risk factors were selected based on the lowest Deviance Information Criterion (DIC) and Watanabe-Akaike Information Criterion (WAIC), with complexity assessed via pDIC and pWAIC [33, 34]. Risk factors from the best-fitting model were considered significant if their 95% credible interval (CrI) did not include zero. To estimate model parameters, the Integrated Nested Laplace Approximation (INLA) package was used. This is a deterministic algorithm known for its efficiency and accuracy in specific spatial and spatiotemporal models [35] in R programming version 2023.06.0. Details of model selection are shown in supplementary document 2.

## Results

### Descriptive analysis of tuberculosis incidence and mortality in Indonesia

**Fig. 1:**
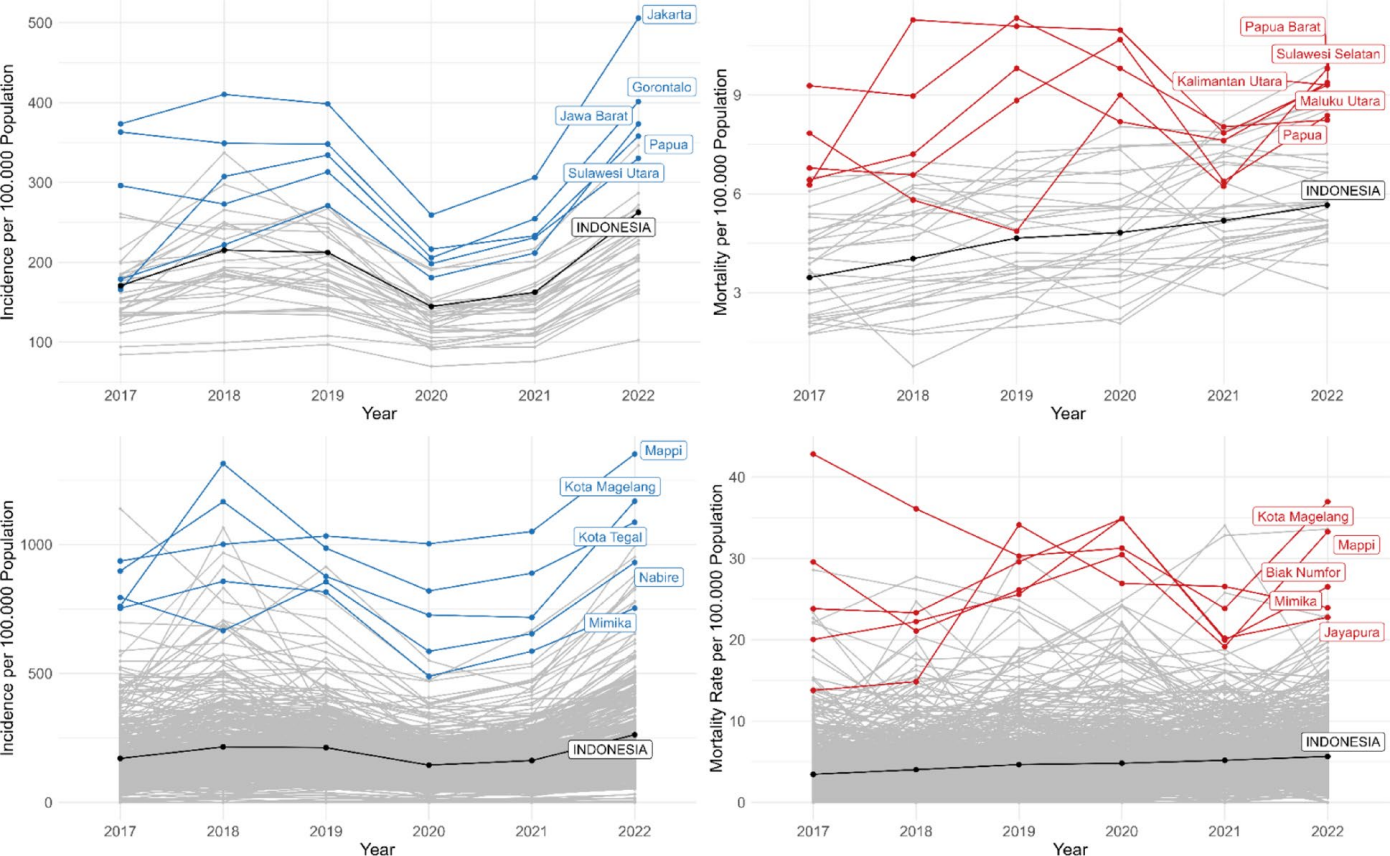
Plots illustrating the trend of the provincial TB incidence per 100,000 population (top-left), provincial TB mortality per 100,000 population (top-right), district TB incidence per 100,000 population (bottom-left), and district TB mortality per 100,000 population (bottom-right)

From 2017 to 2022, there were 3,146,875 total notified TB cases, with 75,071 deaths during treatment. Incidence increased from 2017 to 2018, plateaued in 2019 and decreased in 2020 during the first year of the COVID-19 pandemic followed by a large increase from 2020 to 2022. TB death rates increased steadily over time, started from 3.45 per 100,000 population in 2017 to 5.66 per 100,000 in 2022. At provincial level, the highest mean incidence and mortality rates were both in Papua (Fig. 1). Most provinces showed the same trends over time as the national trend. At district level, Mappi in South Papua Province, and Magelang City in Central Java Province had the highest mean for both TB incidence and mortality (Fig. 1).

**Fig. 2:**
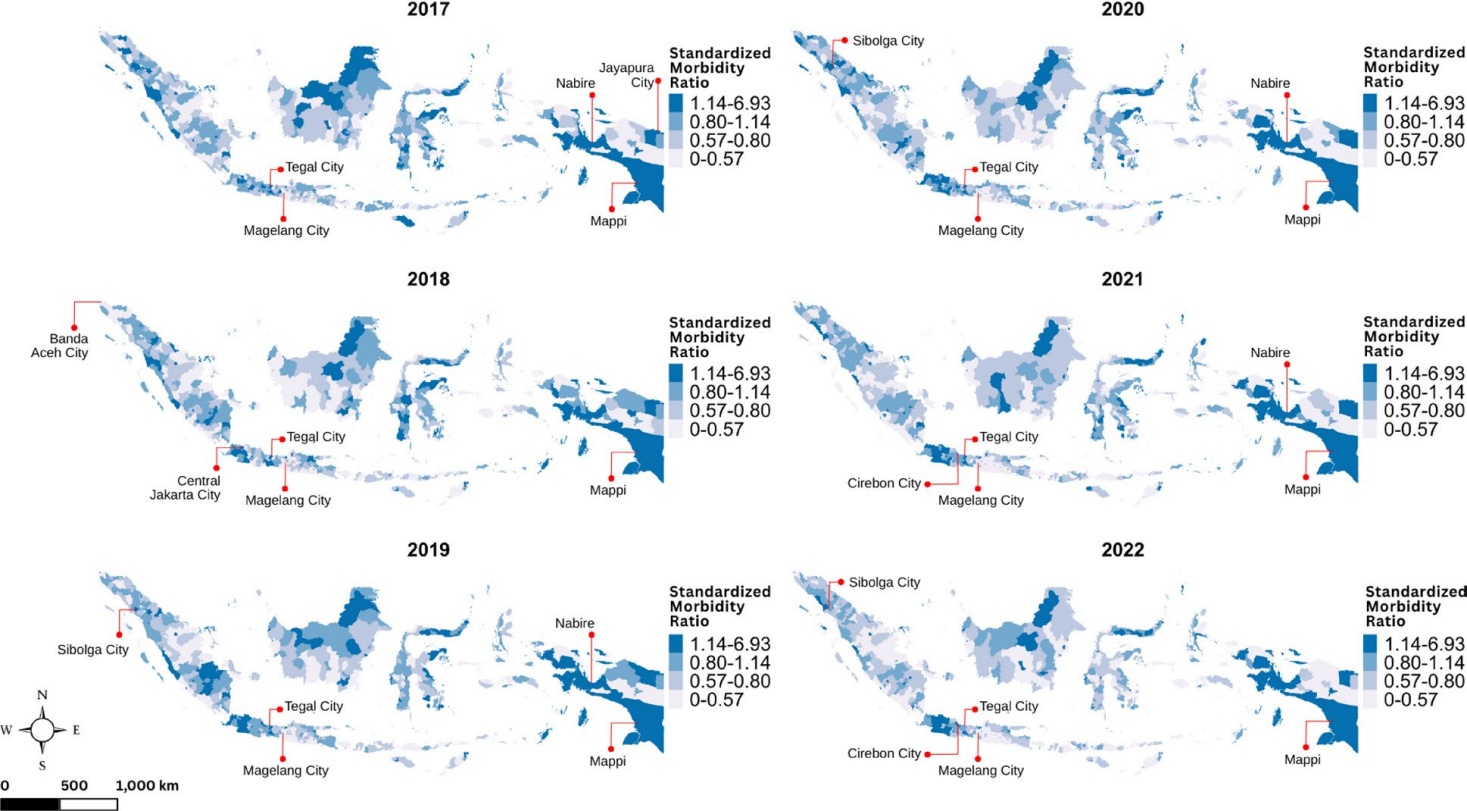
Maps of the district-level TB standardized morbidity ratio in Indonesia during 2017–2022, with labels indicating the top five districts with the highest standardized morbidity ratios

**Fig. 3:**
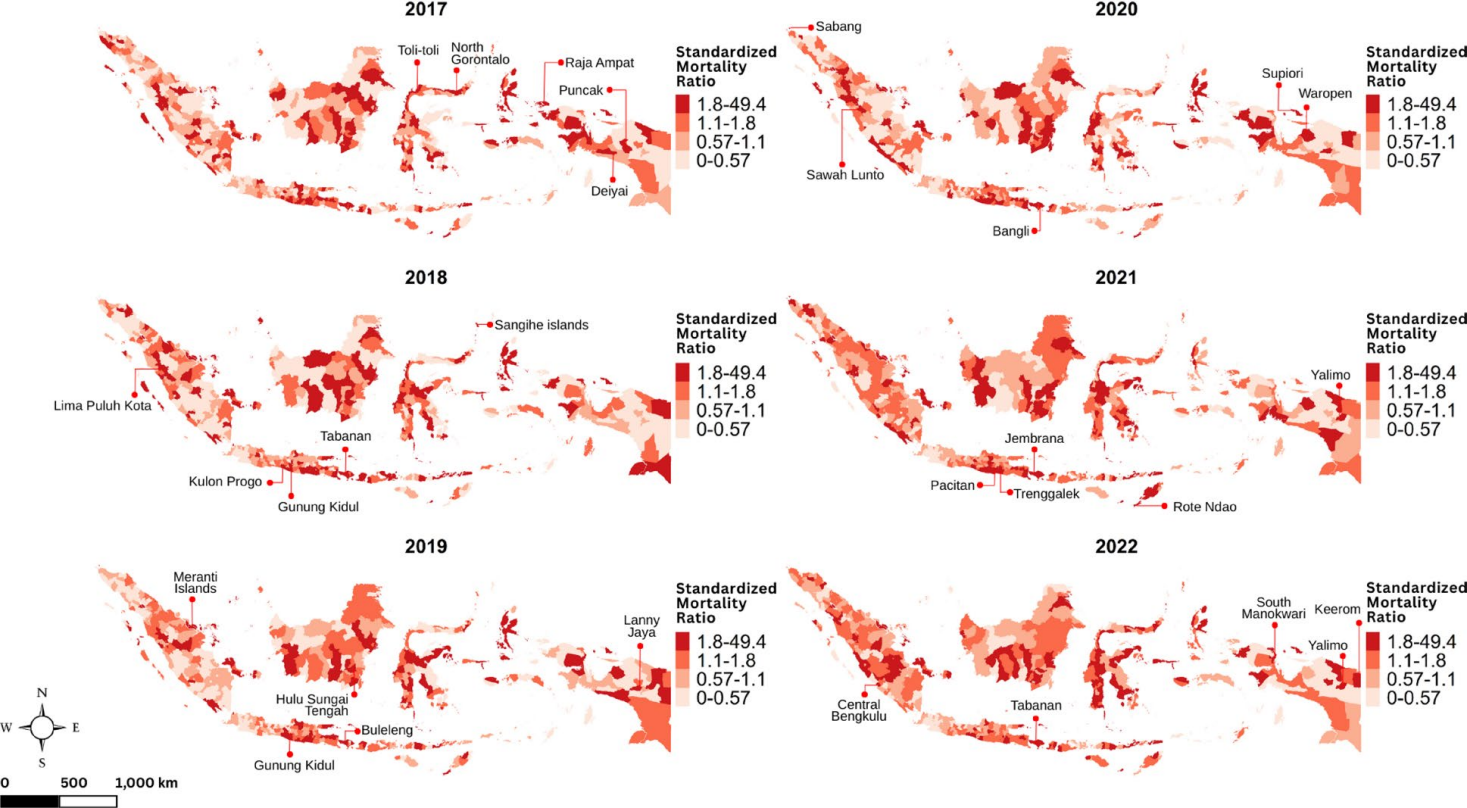
Maps of the district-level TB standardized mortality ratio in Indonesia during 2017–2022, with labels indicating the top five districts with the highest standardized mortality ratios

Figures 2 and 3 display spatiotemporal pattern of the standardized morbidity and mortality ratios respectively. Districts with higher incidence had an $\:{SMR}_{{c}_{it}}$ greater than 1, confirming the observed number of cases exceeded what was expected. Similarly, districts with $\:{SMR}_{{m}_{it}}$ greater than 1 indicate that the number of deaths surpassed what was expected. In terms of geographic distribution, high $\:{SMR}_{{c}_{it}}$values were mainly concentrated in urban areas, particularly in cities such as Tegal City, Magelang City, Central Jakarta City, Sibolga City, Cirebon City, and Mappi. Meanwhile, areas with high $\:{SMR}_{{m}_{it}}$ values were more widely distributed across non-urban and remote island districts, with notable examples including Gunung Kidul, Meranti Islands, Sangihe Islands, Raja Ampat, Rote Ndao, and Keerom. Related to heterogeneity, the spatial variation of $\:{SMR}_{{c}_{it}}$ is more moderate, while variation of $\:{SMR}_{{m}_{it}}\:$ maps is sharper.

**Fig. 4:**
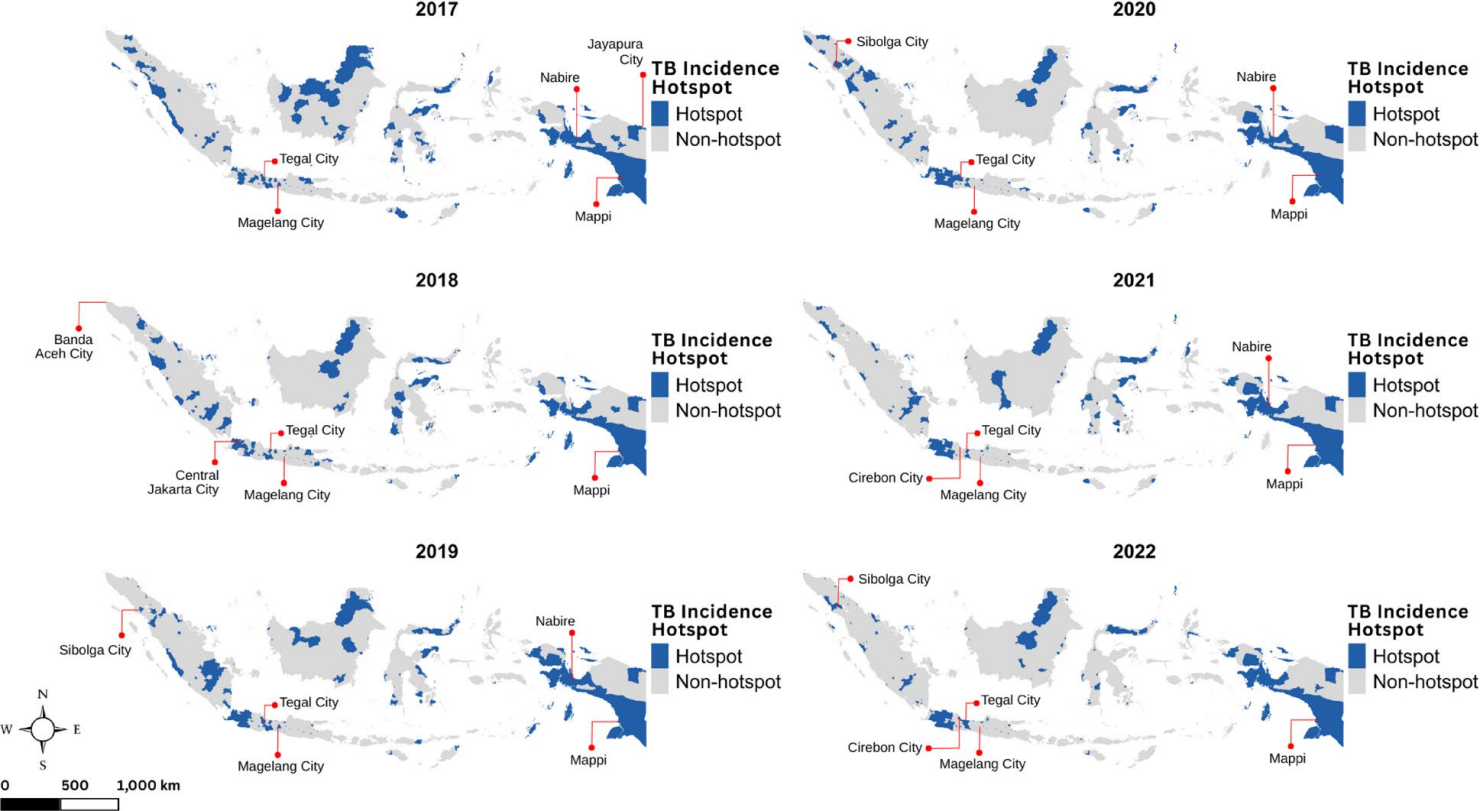
Maps of TB incidence hotspots in Indonesia during 2017–2022, with labels indicating the top five districts with the highest standardized morbidity ratios

**Fig. 5:**
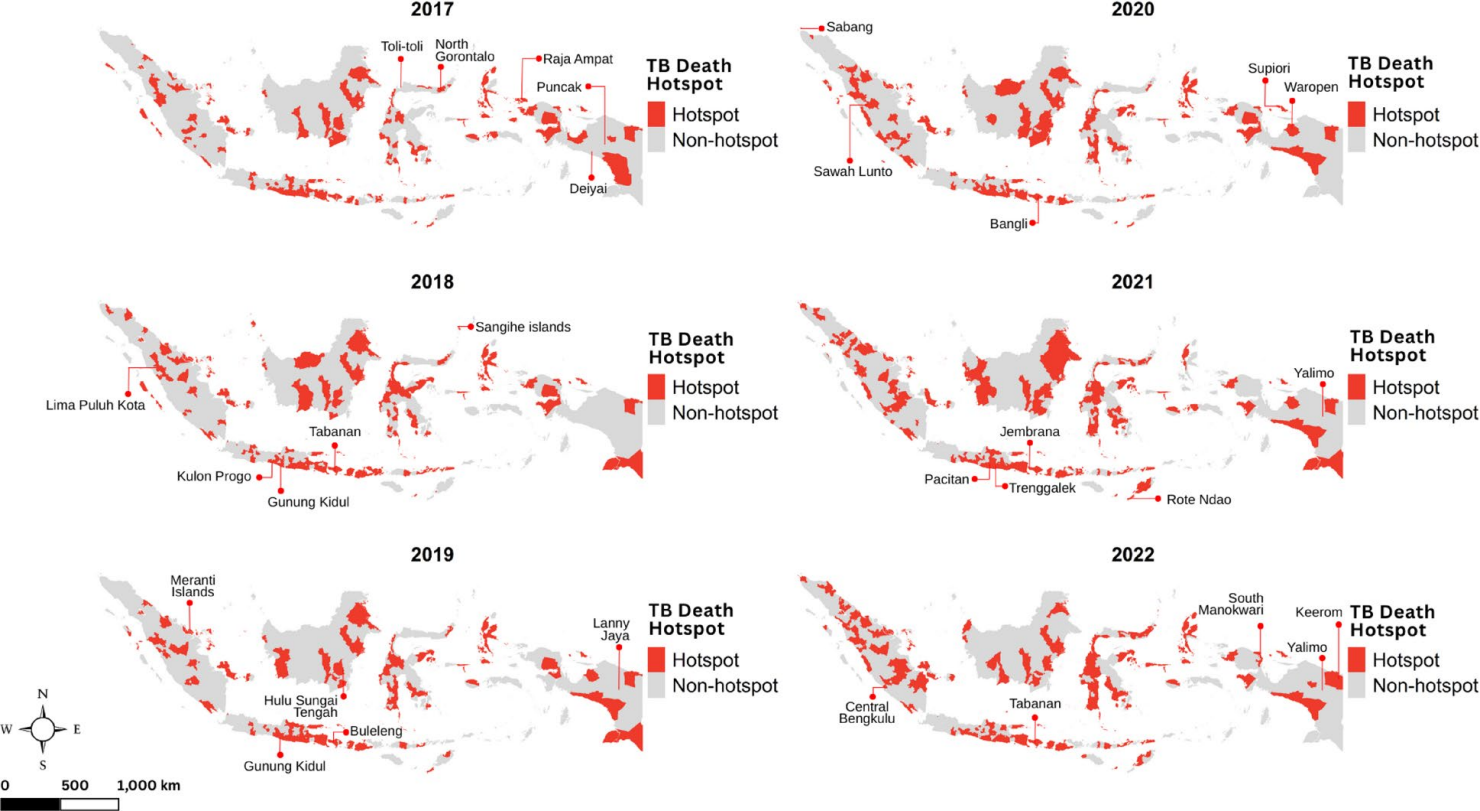
Maps of TB mortality hotspots in Indonesia during 2017–2022, with labels indicating the top five districts with the highest standardized mortality ratios

The most notable information from Fig. 4 was a grouped pattern for TB hotspots, where they were formed in geographically adjacent districts, forming disease cluster or groups of districts. The distribution of TB incidence hotspots closely resembles that of $\:{SMR}_{{c}_{it}}$, where urbanized areas tend to have higher values. On Java Island, the most populous island, TB hotspots were concentrated throughout Jakarta and extended southward into neighbouring districts of West Java Province. Meanwhile, the hotspots distribution for TB mortality tended to be scattered across islands as depicted in Fig. 5. We also noted that the emergence of hotspots increased each year in line with the rising mortality rate, suggesting that as mortality increased, the hotspots became more prominent.

### Model comparison and associations with risk factors

We evaluated and compared multiple modeling configurations to identify spatiotemporal patterns and risk factors associated with TB incidence and TB mortality, using both Poisson and Negative Binomial likelihoods. Full details of the model specifications and evaluation results are provided in supplementary document S3. Table 1 presents the rate ratio (RR) estimates for TB incidence and mortality based on the best-performing models identified through multiple evaluation criteria. Statistically significant risk factors are indicated with asterisks.

For TB incidence, models incorporating the BYM spatial structure, RW1 temporal effects, and type I spatial-temporal interaction demonstrated improved model performance and fit. Based on DIC and WAIC scores, Model 4 under the Poisson likelihood exhibited the best fit among all models tested, outperforming the Negative Binomial alternatives. Although Negative Binomial models had lower penalized complexity (pDIC and pWAIC) scores, Model 4 ultimately provided the best overall balance of fit and parsimony for explaining TB incidence.

Under this model, TB incidence was positively associated with the density of health centers per 100,000 population (RR: 1.007–1.025) and the municipal human development index (RR: 1.049–1.075). These findings suggest that higher access to healthcare infrastructure and higher socioeconomic development levels are associated with greater notification of TB cases, possibly reflecting improved detection and reporting. By contrast, the proportion of the insured population, poverty headcount index, and access to sanitation had 95% credible intervals covering 1, suggesting no strong associations with TB incidence.

In modeling TB mortality, the best-performing model differed by likelihood function. Under the Poisson likelihood, Model 40—which included RW1 temporal effects and type I space-time interaction—demonstrated the lowest DIC and WAIC values. In comparison, Model 56 under the Negative Binomial likelihood also performed reasonably but did not outperform Model 40 in overall goodness-of-fit, despite slightly lower penalized complexity scores. These results suggest that the Poisson model demonstrated greater robustness and reliability for modeling TB mortality risk factors.

Model 40 indicated that treatment-related factors, rather than health system or socioeconomic variables, were significantly associated with TB mortality. Higher treatment coverage (RR: 0.552–0.674) and higher treatment success rates (RR: 0.491–0.721) were both associated with lower mortality, while higher treatment completion rates were associated with increased mortality (RR: 1.860–2.599). No strong associations were observed between TB mortality and district-level DR-TB burden, health center distribution, insurance coverage, poverty rates, sanitation access, or life expectancy at birth.

**Table 1:** Estimates of rate ratios for TB incidence and mortality with their 95% credible intervals based on the best fitted bayesian regression models

Risk factors	Rate ratio estimates (95% CrI)
**Incidence**	
Health centers per 100,000 population	1.016 (1.007, 1.025) *
Universal Health Coverage	1.000 (0.999, 1.001)
Poverty headcount index	0.993 (0.983, 1.002)
Households with access to sanitation	1.000 (0.999, 1.002)
Municipal human development index	1.062 (1.049, 1.075) *
**Mortality**	
TB treatment coverage	0.610 (0.552, 0.674) *
TB treatment completion rate	2.198 (1.860, 2.599) *
TB treatment success rate	0.595 (0.491, 0.721) *
Health centers per 100,000 population	1.003 (0.992, 1.015)
Universal Health Coverage	1.002 (1.001, 1.003)
Poverty headcount index	0.991 (0.982, 1.000)
Households with access to sanitation	1.003 (1.001, 1.005)
Drug-resistant Tuberculosis	0.999 (0.998, 1.001)
Life expectancy at birth	0.992 (0.974, 1.010)

Statistically significant risk factors are indicated with asterisks

## Discussion

This study explored the spatial patterns and risk factors of TB incidence and mortality in Indonesia, which has the second highest TB burden globally. We revealed a significant decline in the numbers of notified TB cases during 2020 and 2021, likely reflecting the disruption in case detection and notification systems due to the COVID-19 pandemic. Interestingly, this decline in reported cases was not mirrored in the mortality rate, suggesting that while TB diagnosis and reporting may have been affected, the impact on deaths among TB patients undergoing treatment was less pronounced. Deaths recorded by the disease control program were only for TB patients who died while they were undergoing TB treatment. This excluded deaths in underdiagnosed TB patients, and those before treatment started, or after treatment ended.

A previous study reported a 26% reduction in the TB case notification rate in Indonesia [36] during COVID-19 pandemic, due to reorganization and prioritization of care service and laboratory testing towards COVID-19 [37]. However, the present study also found that the mortality rate of TB increased over time from 3.45 per 100,000 population in 2017 to be 5.66 per 100,000 in 2022, reflects a worsening situation on the disease burden. WHO confirmed that Indonesia experienced an increase of more than 5% in the estimated number of deaths caused by TB in 2022 compared with 2015 [2]. Another factor may be behind this rise, as the country had the second highest gap between case notifications and estimated total due to underreported cases to the health system and underdiagnosed suspected TB cases [38]. This highlights the challenges of maintaining accurate surveillance during public health crises and underscores the importance of strengthening health systems to ensure the continuity of essential TB services, even during emergencies.

There were differences in the spatial patterns of TB morbidity and mortality hotspots. TB incidence hotspots exhibited a visually clustered pattern, while mortality hotspots were more scattered. We identified TB hotspots in areas with strong population movement, particularly in districts geographically adjacent to Jakarta. This finding aligns with a study from Malaysia using social network analysis (SNA), which found that TB patients who are more central in the mobility network are more likely to influence TB transmission [39]. There was a group of TB hotspots in Southern Papua and North Sulawesi, where the affected districts were adjacent to each other. Similarly, a spatial analysis study in China found that counties with high TB incidence tended to be located next to other high-incidence areas [40]. Meanwhile, unlike the incidence, the spatial distribution of TB deaths is not necessarily influenced by proximity between districts. This contrasts with a study in Brazil, which revealed a clustered pattern of TB-related deaths [41].

The ecological analysis using Bayesian spatial regression modelling revealed that the number of health centers per 100,000 population and the MHDI (Municipal Human Development Index) were significant factors of TB incidence, both showing positive associations. This may imply that areas with more accessible and better-equipped healthcare facilities are likely to detect and notify more cases. Similar findings were reported in a study from southwest Ethiopia [19], suggesting that regions with higher MHDI likely have better access to case detection, stronger health-seeking behaviour, and overall better healthcare infrastructure. Furthermore, country-level analyses have shown that a higher Human Development Index (HDI) is associated with increased testing and TB incidence rates, likely due to improved reporting and surveillance systems [18].

Modelling risk factors for TB mortality at district-level found that only treatment performance outcomes influenced mortality. Mortality was negatively associated with treatment coverage and success rates. Higher coverage means quicker detection and broader access to treatment, as individual level studies confirmed that underdiagnosis, delays in diagnosis, and delays in starting treatment were independent risk factor for death [42, 43]. However, the positive association with treatment completion rates may reflect the weakness of self-reported compliance during prolonged treatment, leading to over-reporting.

The national TB treatment completion rate in Indonesia rose from 43% in 2017 to 63% in 2022, yet this progress may not fully reflect real treatment compliance. Treatment adherence is often based on patients’ self-reports when collecting medications, which can overestimate actual compliance. Because these reports are not objectively verified, underlying non-adherence may go unnoticed, increasing the risk of poor outcomes or death. A study in Mumbai showed that patient-reported adherence was much higher than levels confirmed through urine rifampicin testing, highlighting the limits of self-reported data [44].

Self-reported adherence remains a practical and non-invasive approach, especially in resource-limited settings. However, it mainly serves as an early warning of potential non-adherence and has several limitations. It is prone to social desirability bias, where patients overreport adherence to appear cooperative, and recall bias, when they fail to remember missed doses accurately. In addition, self-reported measures lack timing accuracy, making it difficult to determine when and how consistently medications are taken. Self-reported measures are still useful for monitoring treatment adherence but can be improved by encouraging honest reporting, using scaled responses like anchored Likert scales, defining clear recall periods, and separating data collectors from support staff to reduce bias. Objective checks, such as urine biomarker tests, can also verify adherence [45].

The modelling found that DR-TB, socioeconomic factors (poverty headcount index, household access to sanitation, and life expectancy), and health systems (healthcare access per 100,000 population and UHC) were not associated with mortality. This differs from the positive association found between Tuberculosis death rate and poverty in a study in South Africa which used spatial lag regression [46]. Although TB is commonly referred to as a “disease of poverty”, this can be interpreted as TB incidence and mortality being influenced by multiple overlapping factors beyond poverty alone. There was also found to be no evidence that DR-TB significantly contributes to mortality, consistent with a Global Burden of Disease 2017 study in which most TB deaths occurred in patients with drug-susceptible TB [47].

Beyond treatment, health system, and socioeconomic factors, other contextual determinants identified in similar studies may also influence TB incidence and mortality. In China, TB prevalence was influenced by economic conditions, health investments, air quality, climate, and geography [48]. Similarly, in Ethiopia, higher TB/HIV co-infection rates were linked to low wealth, low literacy, and proximity to borders [49]. An ecological study of 22 WHO high-burden countries found that investments in advocacy and TB–HIV collaboration improved case detection and testing, while funding for drugs, management, and MDR-TB control reduced smear-positive deaths [50]. Another study in Brazil found that areas with higher TB mortality had higher TB incidence, more HIV testing among TB patients, lower education, and more informal jobs [51].

Climatic factors also affect TB and mortality. A study in Brunei, a country with a similar equatorial climate to Indonesia, found that higher minimum temperatures and rainfall increased TB cases after about 30–42 weeks [52]. Although few studies have examined climate effects on TB deaths, a cohort study in China showed that higher PM2.5 levels increased TB mortality by 30% for every 2.06 µg/m³ rise [53] and air pollution levels tend to rise during extreme heat events [54]. Climate change may contribute to long-term increases in TB prevalence by indirectly raising TB risk through six interconnected feedback loops, linking factors such as heatwaves and energy use, indoor time and airborne disease risk, food access and price, malnutrition and infectious disease, healthcare costs and detection delays, and infectious contact and TB risk [55]. Another review showed that changes in temperature, humidity, and rainfall can weaken immunity through reduced vitamin D, malnutrition, and less sunlight exposure, while extreme weather can disrupt TB diagnosis and treatment [56].

The present analysis evaluated models using both Poisson and Negative Binomial likelihoods to address potential overdispersion. Consistent with previous studies on spatiotemporal modeling of surveillance data [57, 58], the Poisson model consistently demonstrated better performance based on goodness-of-fit metrics. This may imply that a Poisson likelihood with appropriate space-time random effects is sufficient to capture the additional variability present in spatiotemporal surveillance data. The adaptability of the Poisson model’s posterior distribution for random effects may contribute to this adequacy, as the prior distribution can effectively adjust to the data’s variability in the posterior distribution. Nonetheless, exploring alternative model specifications with different dispersion distributions could yield further insights.

We acknowledge limitations in this study. First, when interpreting causal inferences, the potential influence of confounders may not have been fully addressed, as the analysis relied on aggregated data, limiting the ability to account for individual-level variability. Second, key comorbidities such as HIV and diabetes mellitus, which are known to significantly increase TB mortality, were not included as covariates due to data unavailability. Finally, the study relied on notified TB data from the National Tuberculosis Surveillance System, which may be subject to underdiagnosis and underreporting, potentially affecting the accuracy of the findings.

## Conclusions

This study investigated TB incidence and mortality down to the district level in Indonesia, which ranks as having the second-highest burden of TB globally and revealed distinct trends, patterns and associated risk factors. TB incidence demonstrated an overall increasing trend, although it fluctuated, with notable disruptions during the COVID-19 pandemic. In contrast, mortality showed a steady upward trajectory. Hotspots of TB cases exhibited spatial groupings, whereas mortality hotspots were more scattered. Incidence increased with better healthcare access and higher MHDI leading to higher notifications, reflecting better surveillance and case reporting concentrated in more developed areas. Conversely, mortality was linked to poor treatment outcomes, such as lower coverage and success rates.

The findings highlighted the need for tailored comprehensive public health strategies of TB treatment, prevention and control to address the distinct drivers of TB morbidity and mortality. Strengthening healthcare access and prioritizing active case finding in high-incidence districts can enhance case detection and promote earlier treatment initiation. To reduce mortality, programmatic efforts should emphasize improving treatment success rates rather than focusing solely on completion rates, ensuring that patients achieve full cure outcomes. Additionally, strengthening adherence monitoring can help mitigate self-reported compliance biases and improve treatment effectiveness.

## Supplementary Information

Supplementary Material 1

## Data Availability

The data supporting the findings of this study were obtained from Indonesia’s National Tuberculosis Program through the Sistem Informasi Tuberkulosis (SITB). Access to these data is restricted and was granted solely for the purposes of this study; therefore, the data are not publicly available.

## Abbreviations

BYM: Besag, york, and mollié
CAR: Conditional autoregressive
CrI: Credible interval
DIC: Deviance information criterion
DR-TB: Drug-resistant TB
INLA: Integrated nested laplace approximation
MHDI: Municipal human development index
NB: Negative binomial
NTSS: National tuberculosis surveillance system
RW1: Random walk of order 1
SNA: Social network analysis
SMR: Standardized morbidity ratios
SMR: Standardized mortality ratios
TB: Tuberculosis
UHC: Universal health coverage
WAIC: Watanabe-akaike information criterion
