# Supplementary material for: Spatiotemporal epidemiology and associated risk factors of tuberculosis incidence and mortality in Indonesia 2017–2022: a nationwide space-time hierarchical analysis
Source: Popul Health Metr. 2026 Jan 31;24:12. doi: 10.1186/s12963-026-00458-5 (PMC12952012; doi:10.1186/s12963-026-00458-5)
Supplement: Supplementary file 1 — Supplementary Material 1 [file 12963_2026_458_MOESM1_ESM.docx]

**Spatiotemporal epidemiology and associated risk factors of tuberculosis incidence and mortality in Indonesia 2017–2022: a nationwide space-time hierarchical analysis**

**Supplementary document S1: Covariates used as potential risk factors in the study and multicollinearity assessment**

**Table S1.1** Summary and explanation of potential risk factors for both TB incidence and mortality.

| **No** | **Covariates** | **Definition** | **TB incidence** | **TB mortality** | **Source** |
| --- | --- | --- | --- | --- | --- |
| 1 | Health center per 100,000 population | Number of public health centers (registered in NTP which serve DR-TB treatment) per 100,000 population | ✓ | ✓ | National Statistical Agency |
| 2 | Universal Health Coverage | Proportion of population that is covered by national health insurance system (contributory and non-contributory schemes) | ✓ | ✓ | National Agency for Health Security |
| 3 | Poverty headcount index | Percentage of the population with expenditure per capita below the poverty line | ✓ | ✓ | National Statistical Agency |
| 4 | Access to sanitation | Percentage of households which have good access to sanitation and are using improved sanitation facilities | ✓ | ✓ | National Statistical Agency |
| 5 | Municipal Human Development Index | A localized version of the Human Development Index (HDI) that quantifies a region’s average achievements in basic human development | ✓ | - | National Statistical Agency |
| 6 | TB treatment coverage | The number of new and relapse cases detected and treated in a given year, divided by the estimated number of incident TB cases in the same year, expressed as a percentage | - | ✓ | Indonesia Ministry of Health |
| 7 | TB treatment complete rate | Proportion of notified TB patients who complete their prescribed course of treatment | - | ✓ | Indonesia Ministry of Health |
| 8 | TB treatment success rate | Proportion of notified TB patients who complete their prescribed course of treatment with no bacteriological evidence of ongoing infection at follow-up | - | ✓ | Indonesia Ministry of Health |
| 9 | Number of Drug-resistant Tuberculosis | Number of TB patients that are resistant to at least one first-line anti-TB drug. | - | ✓ | Indonesia Ministry of Health |
| 10 | Life expectancy at birth (years) | The average number of years that a newborn could expect to live | - | ✓ | National Statistical Agency |


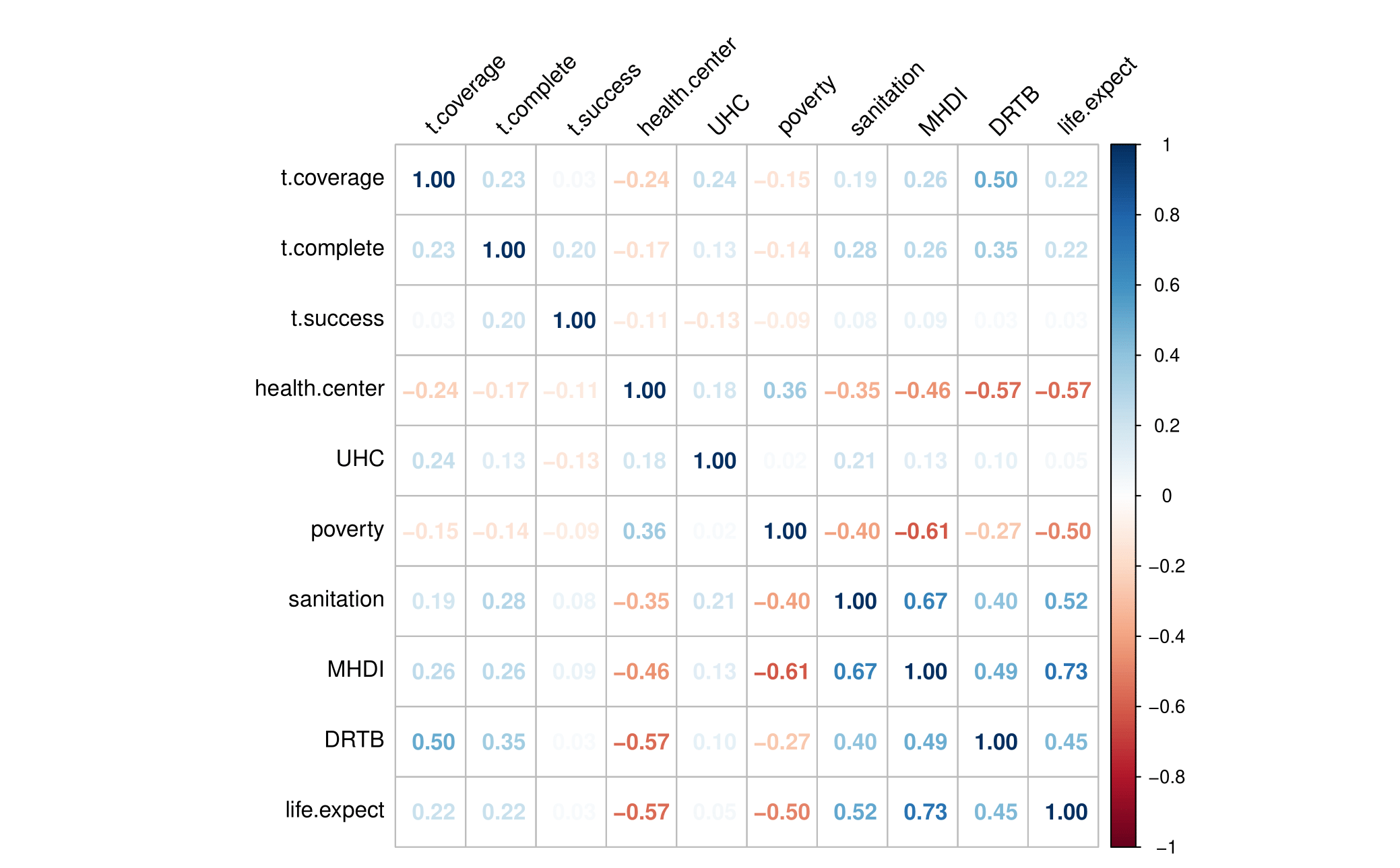


**Figure S1.1** Spearman correlation matrix showing pairwise correlations among study variables.

**Supplementary document S2: Data analysis methods**

*Rate standardization*

For standardized morbidity ratio,

$E_{c_{it}}=\frac{\sum_{t=1}^{T} O_{c_{it}}}{\sum_{t=1}^{T} P_{n_{it}}}*P_{it}$ (1)

${SMR}_{c_{it}}=\frac{O_{c_{it}}}{E_{c_{it}}}$. (2)

For standardized mortality ratio,

$E_{m_{it}}=\frac{\sum_{t=1}^{T} O_{m_{it}}}{\sum_{t=1}^{T} P_{c_{it}}}*P_{c_{it}}$ (3)

${SMR}_{m_{it}}=\frac{O_{m_{it}}}{E_{m_{it}}}.$ (4)

*Spatiotemporal hierarchical Bayesian modeling for TB incidence and mortality*

For TB incidence,

$O_{c_{it}} \sim Poisson \left( \mu_{c_{it},}E_{c_{it}} \right)$ (5)

$\log\left( \mu_{c_{it}} \right)=\log(E_{c_{it}})+\theta_{c_{it}}$ (6)

$\theta_{c_{it}}=\alpha_{0}+\sum_{j=1}^{J} \text{β}\text{j}\text{ X}\text{it}\text{j}+u_{i}+v_{i}+\lambda_{t}^{RW1}+\delta_{it}$ (7)

For TB mortality,

$O_{m_{it}} \sim Poisson \left( \mu_{m_{it},}E_{m_{it}} \right)$ (8)

$\log\left( \mu_{m_{it}} \right)=\log(E_{m_{it}})+\theta_{m_{it}}$ (9)

$\theta_{m_{it}}=\alpha_{0}+\sum_{j=1}^{J} \text{β}\text{j}\text{ X}\text{it}\text{j}+u_{i}+v_{i}+\lambda_{t}^{RW1}+\delta_{it}$ (10)

we incorporated and evaluated the Negative Binomial (NB) likelihood alongside the Poisson likelihood in this study. Therefore, the model likelihood variants were also specified as $O_{c_{it}} \sim Negative Binomial \left( \mu_{c_{it},}E_{c_{it}} \right)$ and $O_{m_{it}} \sim Negative Binomial \left( \mu_{m_{it},}E_{m_{it}} \right)$.

**Supplementary document S3:** Spatiotemporal model specifications, coefficient estimates for each risk factor, and model evaluation results under different model configurations.

**Table S3.1** Modelling TB incidence and risk factor associations in a multivariable Bayesian spatial regression analysis using different structures of random effects and likelihood assumptions

| **No** | **Model specification** | **Risk factors** | | | | | **Evaluation metrices** | |
| --- | --- | --- | --- | --- | --- | --- | --- | --- |
|  |  | **Health centers per 100,000 population** | **Proportion of population with health insurance** | **Population below poverty line** | **Households with access to sanitation** | **Municipal human development index** | **DIC (**$\boldsymbol{p}$**DIC)** | **WAIC (**$\boldsymbol{p}$**WAIC)** |
|  | Poisson likelihood |  |  |  |  |  |  |  |
| 1 | *α_0_ +* $\sum_{j=1}^{J} \text{β}\text{j}\text{. X}\text{it}\text{j}$ *+ u +* $\lambda_{t}^{RW1}$ | 0.009 (0.008 to 0.010) | 0.002 (0.002 to 0.002) | 0.027 (0.026 to 0.028) | -0.001 (-0.001 to -0.001) | 0.069 (0.067 to 0.070) | -47094.61 (-106832.27) | 342255.98 (128621.89) |
| 2 | *α_0_ +* $\sum_{j=1}^{J} \text{β}\text{j}\text{. X}\text{it}\text{j}$ *+ v +* $\lambda_{t}^{RW1}$ | 0.009 (0.008 to 0.010) | 0.002 (0.002 to 0.002) | 0.027 (0.026 to 0.028) | -0.001 (-0.001 to -0.001) | 0.069 (0.067 to 0.070) | -47094.63 (-106832.28) | 342255.97 (128621.89_ |
| 3 | *α_0_ +* $\sum_{j=1}^{J} \text{β}\text{j}\text{. X}\text{it}\text{j}$ *+ u + v +* $\lambda_{t}^{RW1}$ | 0.033 (0.030 to 0.036) | 0.002 (0.002 to 0.002) | 0.039 (0.036 to 0.041) | 0.001 (0.000 to 0.001) | 0.048 (0.043 to 0.053) | -26593.28 (-84208.97) | 301814.04 (111756.80) |
| 4 | *α_0_ +* $\sum_{j=1}^{J} \text{β}\text{j}\text{. X}\text{it}\text{j}$ *+ u + v +* $\lambda_{t}^{RW1}$ *+ δ_it_* | 0.017 (0.008 to 0.025) | 0.000 (0.000 to 0.001) | -0.007 (-0.016 to 0.003) | 0.001 (-0.001 to 0.002) | 0.061 (0.049 to 0.073) | 30714.30 (2872.07) | 30493.53 (1815.95) |
| 5 | *α_0_ +* $\sum_{j=1}^{J} \text{β}\text{j}\text{. X}\text{it}\text{j}$ *+ u +* $\lambda_{t}$ | 0.009 (0.001 to 0.010) | 0.002 (0.002 to 0.002) | 0.027 (0.026 to 0.028) | -0.001 (-0.001 to -0.001) | 0.069 (0.067 to 0.070) | -47099.46 (-106834.69) | 342261.76 (128625.94) |
| 6 | *α_0_ +* $\sum_{j=1}^{J} \text{β}\text{j}\text{. X}\text{it}\text{j}$ *+ v +* $\lambda_{t}$ | 0.033 (0.030 to 0.036) | 0.002 (0.002 to 0.002) | 0.038 (0.036 to 0.040) | 0.001 (0.000 to 0.001) | 0.048 (0.043 to 0.054) | -26615.15 (-84222.81) | 301833.18 (111764.59) |
| 7 | *α_0_ +* $\sum_{j=1}^{J} \text{β}\text{j}\text{. X}\text{it}\text{j}$ *+ u + v +* $\lambda_{t}$ | 0.033 (0.030 to 0.036) | 0.002 (0.002 to 0.002) | 0.038 (0.036 to 0.041) | 0.001 (0.000 to 0.001) | 0.049 (0.043 to 0.054) | -26622.07 (-84226.57) | 302055.46 (111874.50) |
| 8 | *α_0_ +* $\sum_{j=1}^{J} \text{β}\text{j}\text{. X}\text{it}\text{j}$ *+ u + v +* $\lambda_{t}$*+ δ_it_* | 0.017 (0.008 to 0.026) | 0.000 (0.000 to 0.001) | -0.006 (-0.016 to 0.003) | 0.001 (-0.001 to 0.002) | 0.063 (0.050 to 0.075) | 30714.81 (2871.75) | 30495.13 (1816.48) |
| 9 | *α_0_ +* $\sum_{j=1}^{J} \text{β}\text{j}\text{. Xi}\text{t}\text{j}$ *+ u +* $\lambda_{t}^{RW2}$ | 0.009 (0.008 to 0.010) | 0.002 (0.002 to 0.002) | 0.027 (0.026 to 0.028) | -0.001 (-0.001 to -0.001) | 0.069 (0.067 to 0.070) | -46989.97 (-106780.10) | 343452.23 (129217.61) |
| 10 | *α_0_ +* $\sum_{j=1}^{J} \text{β}\text{j}\text{. X}\text{it}\text{j}$ *+ v +* $\lambda_{t}^{RW2}$ | 0.033 (0.030 to 0.036) | 0.002 (0.002 to 0.002) | 0.038 (0.036 to 0.040) | 0.001 (0.000 to 0.001) | 0.049 (0.044 to 0.055) | -26682.62 (-84258.21) | 301673.21 (111677.99) |
| 11 | *α_0_ +* $\sum_{j=1}^{J} \text{β}\text{j}\text{. X}\text{it}\text{j}$*+ u + v +*$\lambda_{t}^{RW2}$ | 0.033 (0.030 to 0.036) | 0.002 (0.002 to 0.002) | 0.038 (0.036 to 0.041) | 0.001 (0.000 to 0.001) | 0.048 (0.043 to 0.054) | -26693.82 (-84261.09) | 301843.37 (111766.84) |
| 12 | *α_0_ +* $\sum_{j=1}^{J} \text{β}\text{j}\text{. X}\text{it}\text{j}$ *+ u + v +* $\lambda_{t}^{RW2}$ *+ δ_it_* | 0.017 (0.008 to 0.026) | 0.000 (0.000 to 0.001) | -0.005 (-0.014 to 0.005) | 0.001 (0.000 to 0.003) | 0.064 (0.052 to 0.076) | 30713.96 (2871.62) | 30495.14 (1816.65) |
| 13 | *α_0_ +* $\sum_{j=1}^{J} \text{β}\text{j}\text{. X}\text{it}\text{j}$ *+ u +* $\lambda_{t}^{RW1}$*+ δ_it_* | 0.021 (0.016 to 0.026) | 0.001 (0.000 to 0.001) | 0.019 (0.015 to 0.023) | 0.000 (-0.001 to 0.001) | 0.084 (0.076 to 0.092) | 30809.45 (2945.33) | 30370.78 (1757.77) |
| 14 | *α_0_ +* $\sum_{j=1}^{J} \text{β}\text{j}\text{. X}\text{it}\text{j}$ *+ v +* $\lambda_{t}^{RW1}$*+ δ_it_* | 0.016 (0.008 to 0.024) | 0.000 (0.000 to 0.001) | -0.008 (-0.017 to 0.000) | 0.001 (-0.001 to 0.002) | 0.056 (0.045 to 0.068) | 30714.58 (2871.54) | 30495.91 (1816.91) |
| 15 | *α_0_ +* $\sum_{j=1}^{J} \text{β}\text{j}\text{. X}\text{it}\text{j}$ *+ u +* $\lambda_{t}$*+ δ_it_* | 0.021 (0.015 to 0.026) | 0.001 (0.000 to 0.001) | 0.019 (0.015 to 0.023) | 0.000 (-0.002 to 0.001) | 0.084 (0.076 to 0.092) | 30808.05 (2945.82) | 30366.14 (1756.23) |
| 16 | *α_0_ +* $\sum_{j=1}^{J} \text{β}\text{j}\text{. X}\text{it}\text{j}$ *+ v +* $\lambda_{t}$*+ δ_it_* | 0.016 (0.008 to 0.024) | 0.000 (0.000 to 0.001) | -0.008 (-0.017 to 0.000) | 0.001 (-0.001 to 0.002) | 0.057 (0.046 to 0.068) | 30714.77 (2871.66) | 30494.75 (1816.29) |
| 17 | *α_0_ +* $\sum_{j=1}^{J} \text{β}\text{j}\text{. X}\text{it}\text{j}$ *+ u +* $\lambda_{t}^{RW2}$*+ δ_it_* | 0.021 (0.015 to 0.026) | 0.000 (0.000 to 0.001) | 0.019 (0.015 to 0.023) | 0.000 (-0.001 to 0.002) | 0.084 (0.076 to 0.092) | 30807.35 (2945.84) | 30365.85 (1756.26) |
| 18 | *α_0_ +* $\sum_{j=1}^{J} \text{β}\text{j}\text{. X}\text{it}\text{j}$ *+ v +* $\lambda_{t}^{RW2}$*+ δ_it_* | 0.016 (0.008 to 0.024) | 0.000 (0.000 to 0.001) | -0.008 (-0.017 to 0.000) | 0.001 (-0.001 to 0.002) | 0.057 (0.046 to 0.069) | 30714.71 (2871.63) | 30494.76 (1816.31) |
|  |  |  |  |  |  |  |  |  |
|  | Negative Binomial likelihood |  |  |  |  |  |  |  |
| 19 | *α_0_ +* $\sum_{j=1}^{J} \text{β}\text{j}\text{. X}\text{it}\text{j}$ *+ u +* $\lambda_{t}^{RW1}$ | 0.023 (0.018 to 0.028) | 0.001 (0.000 to 0.002) | 0.019 (0.015 to 0.023) | 0.000 (-0.001 to 0.001) | 0.057 (0.046 to 0.068) | 40146.35 (473.58) | 40185.67 (453.96) |
| 20 | *α_0_ +* $\sum_{j=1}^{J} \text{β}\text{j}\text{. X}\text{it}\text{j}$ *+ v +* $\lambda_{t}^{RW1}$ | 0.016 (0.008 to 0.024) | 0.000 (0.000 to 0.001) | -0.008 (-0.016 to 0.001) | 0.000 (-0.001 to 0.002) | 0.057 (0.046 to 0.068) | 39106.89 (505.13) | 39115.41 (467.72) |
| 21 | *α_0_ +* $\sum_{j=1}^{J} \text{β}\text{j}\text{. X}\text{it}\text{j}$ *+ u + v +* $\lambda_{t}^{RW1}$ | 0.017 (0.004 to 0.026) | 0.000 (0.000 to 0.001) | -0.005 (-0.014 to 0.004) | 0.000 (-0.001 to 0.002) | 0.063 (0.051 to 0.075) | 39108.07 (505.47) | 39116.43 (467.92) |
| 22 | *α_0_ +* $\sum_{j=1}^{J} \text{β}\text{j}\text{. X}\text{it}\text{j}$ *+ u + v +* $\lambda_{t}^{RW1}$ *+ δ_it_* | 0.017 (0.009 to 0.026) | 0.000 (0.000 to 0.001) | -0.005 (-0.014 to 0.004) | 0.000 (-0.001 to 0.002) | 0.063 (0.051 to 0.075) | 39107.57 (510.29) | 39116.04 (471.85) |
| 23 | *α_0_ +* $\sum_{j=1}^{J} \text{β}\text{j}\text{. X}\text{it}\text{j}$ *+ u +* $\lambda_{t}$ | 0.023 (0.018 to 0.028) | 0.001 (0.000 to 0.002) | 0.019 (0.015 to 0.023) | 0.000 (-0.001 to 0.001) | 0.085 (0.077 to 0.093) | 40147.41 (474.03) | 40186.62 (454.20) |
| 24 | *α_0_ +* $\sum_{j=1}^{J} \text{β}\text{j}\text{. X}\text{it}\text{j}$ *+ v +* $\lambda_{t}$ | 0.016 (0.008 to 0.025) | 0.000 (0.000 to 0.001) | -0.008 (-0.017 to 0.001) | 0.000 (-0.001 to 0.002) | 0.057 (0.046 to 0.068) | 39108.03 (505.51) | 39116.72 (468.09) |
| 25 | *α_0_ +* $\sum_{j=1}^{J} \text{β}\text{j}\text{. X}\text{it}\text{j}$ *+ u + v +* $\lambda_{t}$ | 0.017 (0.009 to 0.026) | 0.000 (0.000 to 0.001) | -0.005 (-0.015 to 0.004) | 0.000 (-0.001 to 0.002) | 0.063 (0.051 to 0.075) | 39110.36 (507.94) | 39117.71 (469.21) |
| 26 | *α_0_ +* $\sum_{j=1}^{J} \text{β}\text{j}\text{. X}\text{it}\text{j}$ *+ u + v +* $\lambda_{t}$*+ δ_it_* | 0.017 (0.009 to 0.026) | 0.000 (0.000 to 0.001) | -0.005 (-0.015 to 0.004) | 0.000 (-0.001 to 0.002) | 0.063 (0.051 to 0.075) | 39109.41 (513.15) | 39117.38 (473.72) |
| 27 | *α_0_ +* $\sum_{j=1}^{J} \text{β}\text{j}\text{. X}\text{it}\text{j}$ *+ u +* $\lambda_{t}^{RW2}$ | 0.023 (0.018 to 0.028) | 0.001 (0.000 to 0.002) | 0.019 (0.015 to 0.023) | 0.000 (-0.001 to 0.002) | 0.085 (0.077 to 0.093) | 40145.62 (472.26) | 40185.07 (453.09) |
| 28 | *α_0_ +* $\sum_{j=1}^{J} \text{β}\text{j}\text{. X}\text{it}\text{j}$ *+ v +* $\lambda_{t}^{RW2}$ | 0.016 (0.008 to 0.024) | 0.000 (0.000 to 0.001) | -0.007 (-0.015 to 0.002) | 0.001 (-0.001 to 0.002) | 0.057 (0.046 to 0.068) | 39106.38 (503.37) | 39114.51 (466.10) |
| 29 | *α_0_ +* $\sum_{j=1}^{J} \text{β}\text{j}\text{. X}\text{it}\text{j}$ *+ u + v +*$\lambda_{t}^{RW2}$ | 0.017 (0.008 to 0.026) | 0.000 (0.000 to 0.001) | -0.004 (-0.013 to 0.005) | 0.001 (-0.001 to 0.002) | 0.064 (0.052 to 0.076) | 39107.37 (503.74) | 39116.11 (466.83) |
| 30 | *α_0_ +* $\sum_{j=1}^{J} \text{β}\text{j}\text{. X}\text{it}\text{j}$ *+ u + v +* $\lambda_{t}^{RW2}$ *+ δ_it_* | 0.017 (0.008 to 0.025) | 0.000 (0.000 to 0.001) | -0.004 (-0.013 to 0.005) | 0.001 (-0.001 to 0.002) | 0.064 (0.052 to 0.076) | 39104.44 (509.63) | 39111.60 (471.30) |
| 31 | *α_0_ +* $\sum_{j=1}^{J} \text{β}\text{j}\text{. X}\text{it}\text{j}$ *+ u +* $\lambda_{t}^{RW1}$*+ δ_it_* | 0.023 (0.018 to 0.028) | 0.001 (0.000 to 0.002) | 0.019 (0.015 to 0.023) | 0.000 (-0.001 to 0.001) | 0.085 (0.077 to 0.093) | 40147.25 (478.19) | 40185.88 (457.15) |
| 32 | *α_0_ +* $\sum_{j=1}^{J} \text{β}\text{j}\text{. X}\text{it}\text{j}$ *+ v +* $\lambda_{t}^{RW1}$*+ δ_it_* | 0.016 (0.008 to 0.024) | 0.000 (0.000 to 0.001) | -0.008 (-0.016 to 0.001) | 0.000 (-0.001 to 0.002) | 0.057 (0.046 to 0.068) | 39106.92 (512.18) | 39113.40 (471.66) |
| 33 | *α_0_ +* $\sum_{j=1}^{J} \text{β}\text{j}\text{. X}\text{it}\text{j}$ *+ u +* $\lambda_{t}$*+ δ_it_* | 0.023 (0.018 to 0.028) | 0.001 (0.000 to 0.002) | 0.019 (0.015 to 0.023) | 0.000 (-0.001 to 0.001) | 0.085 (0.077 to 0.093) | 40148.08 (477.79) | 40186.84 (456.88) |
| 34 | *α_0_ +* $\sum_{j=1}^{J} \text{β}\text{j}\text{. X}\text{it}\text{j}$ *+ v +* $\lambda_{t}$*+ δ_it_* | 0.016 (0.008 to 0.024) | 0.000 (0.000 to 0.001) | -0.008 (-0.017 to 0.001) | 0.000 (-0.001 to 0.002) | 0.057 (0.046 to 0.068) | 39107.60 (514.73) | 39116.71 (475.80) |
| 35 | *α_0_ +* $\sum_{j=1}^{J} \text{β}\text{j}\text{. X}\text{it}\text{j}$ *+ u +* $\lambda_{t}^{RW2}$*+ δ_it_* | 0.023 (0.018 to 0.028) | 0.001 (0.000 to 0.002) | 0.019 (0.015 to 0.023) | 0.000 (-0.001 to 0.002) | 0.085 (0.077 to 0.093) | 40144.63 (473.30) | 40181.09 (451.30) |
| 36 | *α_0_ +* $\sum_{j=1}^{J} \text{β}\text{j}\text{. X}\text{it}\text{j}$ *+ v +* $\lambda_{t}^{RW2}$*+ δ_it_* | 0.016 (-5.160 to -3.517) | 0.000 (0.000 to 0.001) | -0.007 (-0.016 to 0.001) | 0.001 (-0.001 to 0.002) | 0.058 (0.047 to 0.069) | 39103.77 (505.63) | 39110.88 (467.13) |

**Table S3.2** Modelling TB mortality and risk factors associations in a multivariable Bayesian spatial regression analysis using different structures of random effects and likelihood assumptions

| No | Model Specification | Risk factors | | | | | | | | | Evaluation metrices | |
| --- | --- | --- | --- | --- | --- | --- | --- | --- | --- | --- | --- | --- |
|  |  | Treatment coverage | Treatment completion rate | Treatment success rate | Health centers per 100,000 population | Proportion of population with health insurance | Population below poverty line | Households with access to sanitation | Drug-resistant Tuberculosis | Life expectancy at birth | **DIC (**$p$DIC**)** | **WAIC (**$p$WAIC**)** |
|  | Poisson likelihood |  |  |  |  |  |  |  |  |  |  |  |
| 37 | *α_0_ +* $\sum_{j=1}^{J} \text{β}\text{j}\text{. X}\text{it}\text{j}$ *+ u +* $\lambda_{t}^{RW1}$ | -0.405 (-0.445 to -0.365) | 0.615 (0.530 to 0.700) | -0.636 (-0.735 to -0.537) | -0.009 (-0.016 to -0.002) | 0.001 (0.001 to 0.002) | 0.006 (0.002 to 0.010) | 0.005 (0.004 to 0.007) | 0.000 (0.000 to 0.000) | -0.016 (-0.025 to -0.007) | 24798.74 (85.97) | 29817.52 (3146.06) |
| 38 | *α_0_ +* $\sum_{j=1}^{J} \text{β}\text{j}\text{. X}\text{it}\text{j}$ *+ v +* $\lambda_{t}^{RW1}$ | -0.380 (-0.421 to -0.338) | 0.782 (0.695 to 0.870) | -0.704 (-0.808 to -0.601) | 0.005 (-0.004 to 0.014) | 0.000 (-0.001 to 0.000) | -0.008 (-0.016 to 0.000) | 0.001 (0.000 to 0.003) | 0.000 (0.000 to 0.000) | 0.006 (-0.007 to 0.020) | 23568.85 (186.09) | 27768.76 (2754.67) |
| 39 | *α_0_ +* $\sum_{j=1}^{J} \text{β}\text{j}\text{. X}\text{it}\text{j}$ *+ u + v +* $\lambda_{t}^{RW1}$ | -0.373 (-0.414 to -0.331) | 0.783 (0.696 to 0.871) | -0.710 (-0.813 to -0.606) | 0.007 (-0.003 to 0.017) | 0.000 (-0.001 to 0.000) | -0.011 (-0.018 to -0.003) | 0.001 (0.000 to 0.002) | 0.000 (0.000 to 0.000) | 0.005 (-0.009 to 0.018) | 23571.83 (185.21) | 27848.72 (2792.74) |
| 40 | *α_0_ +* $\sum_{j=1}^{J} \text{β}\text{j}\text{. X}\text{it}\text{j}$ *+ u + v +* $\lambda_{t}^{RW1}$ *+ δ_it_* | -0.493 (-0.593 to -0.394) | 0.788 (0.621 to 0.955) | -0.518 (-0.710 to -0.326) | 0.004 (-0.008 to 0.015) | 0.001 (0.000 to 0.002) | -0.008 (-0.018 to 0.001) | 0.003 (0.000 to 0.005) | 0.000 (-0.001 to 0.001) | -0.008 (-0.026 to 0.010) | 18700.21 (2131.13) | 18632.82 (1519.87) |
| 41 | *α_0_ +* $\sum_{j=1}^{J} \text{β}\text{j}\text{. X}\text{it}\text{j}$ *+ u +* $\lambda_{t}$ | 0.000 (-0.005 to 0.006) | 0.002 (-0.011 to 0.014) | -0.001 (-0.016 to 0.014) | 0.000 (-0.001 to 0.001) | 0.000 (0.000 to 0.000) | 0.000 (-0.001 to 0.001) | 0.000 (0.000 to 0.000) | 0.000 (0.000 to 0.000) | 0.000 (-0.001 to 0.001) | 25221.60 (156.04) | 28498.13 (1683.92) |
| 42 | *α_0_ +* $\sum_{j=1}^{J} \text{β}\text{j}\text{. X}\text{it}\text{j}$ *+ v +* $\lambda_{t}$ | 0.000 (-0.005 to 0.005) | 0.002 (-0.011 to 0.015) | -0.001 (-0.016 to 0.014) | 0.000 (-0.001 to 0.001) | 0.000 (0.000 to 0.000) | 0.000 (-0.001 to 0.001) | 0.000 (0.000 to 0.000) | 0.000 (0.000 to 0.000) | 0.000 (-0.001 to 0.001) | 25211.56 (154.65) | 28265.11 (1583.66) |
| 43 | *α_0_ +* $\sum_{j=1}^{J} \text{β}\text{j}\text{. X}\text{it}\text{j}$ *+ u + v +* $\lambda_{t}$ | 0.000 (-0.005 to 0.006) | 0.002 (-0.011 to 0.015) | -0.001 (-0.016 to 0.014) | 0.000 (-0.001 to 0.001) | 0.000 (0.000 to 0.000) | 0.000 (-0.001 to 0.001) | 0.000 (0.000 to 0.000) | 0.000 (0.000 to 0.000) | 0.000 (-0.002 to 0.001) | 25259.58 (184.48) | 28087.50 (1487.25) |
| 44 | *α_0_ +* $\sum_{j=1}^{J} \text{β}\text{j}\text{. X}\text{it}\text{j}$ *+ u + v +* $\lambda_{t}$*+ δ_it_* | 0.000 (-0.005 to 0.006) | 0.002 (-0.011 to 0.015) | -0.001 (-0.016 to 0.014) | 0.000 (-0.001 to 0.001) | 0.000 (0.000 to 0.000) | 0.000 (-0.001 to 0.001) | 0.000 (0.000 to 0.000) | 0.000 (0.000 to 0.000) | 0.000 (-0.002 to 0.001) | 25299.52 (205.78) | 28097.63 (1483.74) |
| 45 | *α_0_ +* $\sum_{j=1}^{J} \text{β}\text{j}\text{. Xi}\text{t}\text{j}$ *+ u +* $\lambda_{t}^{RW2}$ | 0.000 (-0.005 to 0.006) | 0.002 (-0.011 to 0.014) | -0.001 (-0.016 to 0.014) | 0.000 (-0.001 to 0.001) | 0.000 (0.000 to 0.000) | 0.000 (-0.001 to 0.001) | 0.000 (0.000 to 0.000) | 0.000 (0.000 to 0.000) | 0.000 (-0.001 to 0.001) | 25221.42 (155.89) | 28498.02 (1683.88) |
| 46 | *α_0_ +* $\sum_{j=1}^{J} \text{β}\text{j}\text{. X}\text{it}\text{j}$ *+ v +* $\lambda_{t}^{RW2}$ | 0.000 (-0.005 to 0.005) | 0.002 (-0.011 to 0.015) | -0.001 (-0.016 to 0.014) | 0.000 (-0.001 to 0.001) | 0.000 (0.000 to 0.000) | 0.000 (-0.001 to 0.001) | 0.000 (0.000 to 0.000) | 0.000 (0.000 to 0.000) | 0.000 (-0.001 to 0.001) | 25211.47 (154.54) | 28264.66 (1583.43) |
| 47 | *α_0_ +* $\sum_{j=1}^{J} \text{β}\text{j}\text{. X}\text{it}\text{j}$*+ u + v +*$\lambda_{t}^{RW2}$ | 0.000 (-0.005 to 0.006) | 0.002 (-0.011 to 0.015) | -0.001 (-0.016 to 0.014) | 0.000 (-0.001 to 0.001) | 0.000 (0.000 to 0.000) | 0.000 (-0.001 to 0.001) | 0.000 (0.000 to 0.000) | 0.000 (0.000 to 0.000) | 0.000 (-0.002 to 0.001) | 25259.19 (184.11) | 28092.96 (1490.01) |
| 48 | *α_0_ +* $\sum_{j=1}^{J} \text{β}\text{j}\text{. X}\text{it}\text{j}$ *+ u + v +* $\lambda_{t}^{RW2}$ *+ δ_it_* | 0.000 (-0.005 to 0.006) | 0.002 (-0.011 to 0.015) | -0.001 (-0.017 to 0.014) | 0.000 (-0.001 to 0.001) | 0.000 (0.000 to 0.000) | 0.000 (-0.001 to 0.001) | 0.000 (0.000 to 0.000) | 0.000 (0.000 to 0.000) | 0.000 (-0.002 to 0.001) | 25299.52 (205.79) | 28097.13 (1483.49) |
| 49 | *α_0_ +* $\sum_{j=1}^{J} \text{β}\text{j}\text{. X}\text{it}\text{j}$ *+ u +* $\lambda_{t}^{RW1}$*+ δ_it_* | 0.000 (-0.005 to 0.006) | 0.002 (-0.011 to 0.015) | -0.001 (-0.016 to 0.014) | 0.000 (-0.001 to 0.001) | 0.000 (0.000 to 0.000) | 0.000 (-0.001 to 0.001) | 0.000 (0.000 to 0.000) | 0.000 (0.000 to 0.000) | 0.000 (-0.001 to 0.001) | 25241.00 (170.75) | 28295.94 (1590.58) |
| 50 | *α_0_ +* $\sum_{j=1}^{J} \text{β}\text{j}\text{. X}\text{it}\text{j}$ *+ v +* $\lambda_{t}^{RW1}$*+ δ_it_* | 0.000 (-0.005 to 0.006) | 0.002 (-0.011 to 0.015) | -0.001 (-0.016 to 0.014) | 0.000 (-0.001 to 0.001) | 0.000 (0.000 to 0.000) | 0.000 (-0.001 to 0.001) | 0.000 (0.000 to 0.000) | 0.000 (0.000 to 0.000) | 0.000 (-0.001 to 0.001) | 25241.00 (170.75) | 28295.92 (1590.57) |
| 51 | *α_0_ +* $\sum_{j=1}^{J} \text{β}\text{j}\text{. X}\text{it}\text{j}$ *+ u +* $\lambda_{t}$*+ δ_it_* | 0.000 (-0.005 to 0.006) | 0.002 (-0.011 to 0.015) | -0.001 (-0.016 to 0.014) | 0.000 (-0.001 to 0.001) | 0.000 (0.000 to 0.000) | 0.000 (-0.001 to 0.001) | 0.000 (0.000 to 0.000) | 0.000 (0.000 to 0.000) | 0.000 (-0.001 to 0.001) | 25260.34 (176.59) | 28507.47 (1680.08) |
| 52 | *α_0_ +* $\sum_{j=1}^{J} \text{β}\text{j}\text{. X}\text{it}\text{j}$ *+ v +* $\lambda_{t}$*+ δ_it_* | 0.000 (-0.005 to 0.006) | 0.002 (-0.011 to 0.015) | -0.001 (-0.016 to 0.014) | 0.000 (-0.001 to 0.001) | 0.000 (0.000 to 0.000) | 0.000 (-0.001 to 0.001) | 0.000 (0.000 to 0.000) | 0.000 (0.000 to 0.000) | 0.000 (-0.001 to 0.001) | 25240.98 (170.76) | 28295.32 (1590.25) |
| 53 | *α_0_ +* $\sum_{j=1}^{J} \text{β}\text{j}\text{. X}\text{it}\text{j}$ *+ u +* $\lambda_{t}^{RW2}$*+ δ_it_* | 0.000 (-0.005 to 0.006) | 0.002 (-0.011 to 0.015) | -0.001 (-0.016 to 0.014) | 0.000 (-0.001 to 0.001) | 0.000 (0.000 to 0.000) | 0.000 (-0.001 to 0.001) | 0.000 (0.000 to 0.000) | 0.000 (0.000 to 0.000) | 0.000 (-0.001 to 0.001) | 25258.15 (175.72) | 28477.70 (1665.11) |
| 54 | *α_0_ +* $\sum_{j=1}^{J} \text{β}\text{j}\text{. X}\text{it}\text{j}$ *+ v +* $\lambda_{t}^{RW2}$*+ δ_it_* | 0.000 (-0.005 to 0.006) | 0.002 (-0.011 to 0.015) | -0.001 (-0.016 to 0.014) | 0.000 (-0.001 to 0.001) | 0.000 (0.000 to 0.000) | 0.000 (-0.001 to 0.001) | 0.000 (0.000 to 0.000) | 0.000 (0.000 to 0.000) | 0.000 (-0.001 to 0.001) | 25241.10 (170.93) | 28292.26 (1588.66) |
|  |  |  |  |  |  |  |  |  |  |  |  |  |
|  | Negative Binomial likelihood |  |  |  |  |  |  |  |  |  |  |  |
| 55 | *α_0_ +* $\sum_{j=1}^{J} \text{β}\text{j}\text{. X}\text{it}\text{j}$ *+ u +* $\lambda_{t}^{RW1}$ | -0.541 (-0.639 to -0.444) | 0.607 (0.435 to 0.781) | -0.579 (-0.778 to -0.380) | 0.007 (-0.002 to 0.016) | 0.002 (0.001 to 0.003) | -0.002 (-0.008 to 0.004) | 0.007 (0.005 to 0.009) | 0.000 (-0.001 to 0.001) | -0.002 (-0.017 to 0.013) | 21354.64 (368.44) | 21377.45 (349.70) |
| 56 | *α_0_ +* $\sum_{j=1}^{J} \text{β}\text{j}\text{. X}\text{it}\text{j}$ *+ v +* $\lambda_{t}^{RW1}$ | -0.523 (-0.619 to -0.427) | 0.772 (0.605 to 0.940) | -0.538 (-0.734 to -0.342) | 0.002 (-0.009 to 0.012) | 0.001 (0.000 to 0.002) | -0.006 (-0.014 to 0.003) | 0.004 (0.001 to 0.006) | 0.000 (-0.001 to 0.000) | -0.006 (-0.023 to 0.011) | 20816.23 (436.10) | 20859.66 (422.26) |
| 57 | *α_0_ +* $\sum_{j=1}^{J} \text{β}\text{j}\text{. X}\text{it}\text{j}$ *+ u + v +* $\lambda_{t}^{RW1}$ | -0.484 (-0.580 to -0.389) | 0.768 (0.601 to 0.936) | -0.544 (-0.741 to -0.348) | 0.004 (-0.007 to 0.016) | 0.001 (0.000 to 0.002) | -0.008 (-0.018 to 0.001) | 0.003 (0.001 to 0.005) | 0.000 (-0.001 to 0.001) | -0.010 (-0.027 to 0.008) | 20819.60 (436.04) | 20866.17 (424.62) |
| 58 | *α_0_ +* $\sum_{j=1}^{J} \text{β}\text{j}\text{. X}\text{it}\text{j}$ *+ u + v +* $\lambda_{t}^{RW1}$ *+ δ_it_* | -0.485 (-0.581 to -0.388) | 0.769 (0.602 to 0.938) | -0.544 (-0.741 to -0.348) | 0.004 (-0.007 to 0.016) | 0.001 (0.000 to 0.002) | -0.008 (-0.018 to 0.001) | 0.003 (0.001 to 0.005) | 0.000 (-0.001 to 0.001) | -0.009 (-0.027 to 0.008) | 20818.72 (437.38) | 20865.91 (426.12) |
| 59 | *α_0_ +* $\sum_{j=1}^{J} \text{β}\text{j}\text{. X}\text{it}\text{j}$ *+ u +* $\lambda_{t}$ | 0.000 (-0.005 to 0.006) | 0.002 (-0.011 to 0.014) | -0.001 (-0.016 to 0.014) | 0.000 (-0.001 to 0.001) | 0.000 (0.000 to 0.000) | 0.000 (-0.001 to 0.001) | 0.000 (0.000 to 0.000) | 0.000 (0.000 to 0.000) | 0.000 (-0.001 to 0.001) | 25238.29 (163.56) | 28547.83 (1707.13) |
| 60 | *α_0_ +* $\sum_{j=1}^{J} \text{β}\text{j}\text{. X}\text{it}\text{j}$ *+ v +* $\lambda_{t}$ | 0.000 (-0.005 to 0.005) | 0.002 (-0.011 to 0.015) | -0.001 (-0.016 to 0.014) | 0.000 (-0.001 to 0.001) | 0.000 (0.000 to 0.000) | 0.000 (-0.001 to 0.001) | 0.000 (0.000 to 0.000) | 0.000 (0.000 to 0.000) | 0.000 (-0.001 to 0.001) | 25201.83 (149.53) | 28285.73 (1593.85) |
| 61 | *α_0_ +* $\sum_{j=1}^{J} \text{β}\text{j}\text{. X}\text{it}\text{j}$ *+ u + v +* $\lambda_{t}$ | 0.000 (-0.005 to 0.006) | 0.002 (-0.011 to 0.015) | -0.001 (-0.016 to 0.014) | 0.000 (-0.001 to 0.001) | 0.000 (0.000 to 0.000) | 0.000 (-0.001 to 0.001) | 0.000 (0.000 to 0.000) | 0.000 (0.000 to 0.000) | 0.000 (-0.002 to 0.001) | 25258.53 (183.19) | 28104.80 (1494.78) |
| 62 | *α_0_ +* $\sum_{j=1}^{J} \text{β}\text{j}\text{. X}\text{it}\text{j}$ *+ u + v +* $\lambda_{t}$*+ δ_it_* | 0.000 (-0.005 to 0.006) | 0.002 (-0.011 to 0.015) | -0.001 (-0.016 to 0.014) | 0.000 (-0.001 to 0.001) | 0.000 (0.000 to 0.000) | 0.000 (-0.001 to 0.001) | 0.000 (0.000 to 0.000) | 0.000 (0.000 to 0.000) | 0.000 (-0.002 to 0.001) | 25302.14 (207.10) | 28070.39 (1469.73) |
| 63 | *α_0_ +* $\sum_{j=1}^{J} \text{β}\text{j}\text{. Xi}\text{t}\text{j}$ *+ u +* $\lambda_{t}^{RW2}$ | 0.000 (-0.005 to 0.006) | 0.002 (-0.011 to 0.015) | -0.001 (-0.016 to 0.014) | 0.000 (-0.001 to 0.001) | 0.000 (0.000 to 0.000) | 0.000 (-0.001 to 0.001) | 0.000 (0.000 to 0.000) | 0.000 (0.000 to 0.000) | 0.000 (-0.001 to 0.001) | 25223.99 | 28470.61 |
| 64 | *α_0_ +* $\sum_{j=1}^{J} \text{β}\text{j}\text{. X}\text{it}\text{j}$ *+ v +* $\lambda_{t}^{RW2}$ | 0.000 (-0.005 to 0.005) | 0.002 (-0.011 to 0.015) | -0.001 (-0.016 to 0.014) | 0.000 (-0.001 to 0.001) | 0.000 (0.000 to 0.000) | 0.000 (-0.001 to 0.001) | 0.000 (0.000 to 0.000) | 0.000 (0.000 to 0.000) | 0.000 (-0.002 to 0.001) | 25203.81 (151.03) | 28271.60 (1586.55) |
| 65 | *α_0_ +* $\sum_{j=1}^{J} \text{β}\text{j}\text{. X}\text{it}\text{j}$*+ u + v +*$\lambda_{t}^{RW2}$ | 0.000 (-0.005 to 0.006) | 0.002 (-0.011 to 0.015) | -0.001 (-0.016 to 0.014) | 0.000 (-0.001 to 0.001) | 0.000 (0.000 to 0.000) | 0.000 (-0.001 to 0.001) | 0.000 (0.000 to 0.000) | 0.000 (0.000 to 0.000) | 0.000 (-0.002 to 0.001) | 25259.60 (184.88) | 28076.43 (1481.33) |
| 66 | *α_0_ +* $\sum_{j=1}^{J} \text{β}\text{j}\text{. X}\text{it}\text{j}$ *+ u + v +* $\lambda_{t}^{RW2}$ *+ δ_it_* | 0.000 (-0.005 to 0.006) | 0.002 (-0.011 to 0.015) | -0.001 (-0.017 to 0.014) | 0.000 (-0.001 to 0.001) | 0.000 (0.000 to 0.000) | 0.000 (-0.001 to 0.001) | 0.000 (0.000 to 0.000) | 0.000 (0.000 to 0.000) | 0.000 (-0.002 to 0.001) | 25301.77 (207.06) | 28074.73 (1471.81) |
| 67 | *α_0_ +* $\sum_{j=1}^{J} \text{β}\text{j}\text{. X}\text{it}\text{j}$ *+ u +* $\lambda_{t}^{RW1}$*+ δ_it_* | 0.000 (-0.005 to 0.006) | 0.002 (-0.011 to 0.015) | -0.001 (-0.016 to 0.014) | 0.000 (-0.001 to 0.001) | 0.000 (0.000 to 0.000) | 0.000 (-0.001 to 0.001) | 0.000 (0.000 to 0.000) | 0.000 (0.000 to 0.000) | 0.000 (-0.001 to 0.001) | 25250.48 (171.58) | 28481.89 (1669.22) |
| 68 | *α_0_ +* $\sum_{j=1}^{J} \text{β}\text{j}\text{. X}\text{it}\text{j}$ *+ v +* $\lambda_{t}^{RW1}$*+ δ_it_* | 0.000 (-0.005 to 0.006) | 0.002 (-0.011 to 0.015) | -0.001 (-0.016 to 0.014) | 0.000 (-0.001 to 0.001) | 0.000 (0.000 to 0.000) | 0.000 (-0.001 to 0.001) | 0.000 (0.000 to 0.000) | 0.000 (0.000 to 0.000) | 0.000 (-0.001 to 0.001) | 25250.53 | 28481,86 |
| 69 | *α_0_ +* $\sum_{j=1}^{J} \text{β}\text{j}\text{. X}\text{it}\text{j}$ *+ u +* $\lambda_{t}$*+ δ_it_* | 0.000 (-0.005 to 0.006) | 0.002 (-0.011 to 0.015) | -0.001 (-0.016 to 0.014) | 0.000 (-0.001 to 0.001) | 0.000 (0.000 to 0.000) | 0.000 (-0.001 to 0.001) | 0.000 (0.000 to 0.000) | 0.000 (0.000 to 0.000) | 0.000 (-0.001 to 0.001) | 25252.00 (172.49) | 28404.46 (1627.93) |
| 70 | *α_0_ +* $\sum_{j=1}^{J} \text{β}\text{j}\text{. X}\text{it}\text{j}$ *+ v +* $\lambda_{t}$*+ δ_it_* | 0.000 (-0.005 to 0.006) | 0.002 (-0.011 to 0.015) | -0.001 (-0.016 to 0.014) | 0.000 (-0.001 to 0.001) | 0.000 (0.000 to 0.000) | 0.000 (-0.001 to 0.001) | 0.000 (0.000 to 0.000) | 0.000 (0.000 to 0.000) | 0.000 (-0.001 to 0.001) | 24243,21 | 28115,61 |
| 71 | *α_0_ +* $\sum_{j=1}^{J} \text{β}\text{j}\text{. X}\text{it}\text{j}$ *+ u +* $\lambda_{t}^{RW2}$*+ δ_it_* | 0.000 (-0.005 to 0.006) | 0.002 (-0.011 to 0.015) | -0.001 (-0.016 to 0.014) | 0.000 (-0.001 to 0.001) | 0.000 (0.000 to 0.000) | 0.000 (-0.001 to 0.001) | 0.000 (0.000 to 0.000) | 0.000 (0.000 to 0.000) | 0.000 (-0.001 to 0.001) | 25260.39 (176.56) | 28337.09 (1592.76) |
| 72 | *α_0_ +* $\sum_{j=1}^{J} \text{β}\text{j}\text{. X}\text{it}\text{j}$ *+ v +* $\lambda_{t}^{RW2}$*+ δ_it_* | 0.000 (-0.005 to 0.006) | 0.002 (-0.011 to 0.015) | -0.001 (-0.016 to 0.014) | 0.000 (-0.001 to 0.001) | 0.000 (0.000 to 0.000) | 0.000 (-0.001 to 0.001) | 0.000 (0.000 to 0.000) | 0.000 (0.000 to 0.000) | 0.000 (-0.001 to 0.001) | 25243,33 | 28152,56 |
